# Evaluation of quality of sleep in women with stress urinary incontinence before and after surgical correction

**DOI:** 10.1590/S1679-45082018AO4205

**Published:** 2018-06-21

**Authors:** Josyandra Paula de Freitas, Mariana Pereira Inácio Silvestri, César Eduardo Fernandes, Emerson de Oliveira

**Affiliations:** 1 Faculdade de Medicina do ABC, Santo André, SP, Brazil

**Keywords:** Urinary incontinence/surgery, Sleep wake disorders, Sleep, Women, Incontinência urinária/cirurgia, Transtornos do sono-vigília, Sono, Mulheres

## Abstract

**Objective:**

To evaluate the quality of sleep in women with urinary incontinence before and after sling surgery.

**Methods:**

A prospective study of case series of women with urodynamic diagnosis of stress urinary incontinence. To evaluate the subjective quality of sleep, two specific questionnaires were used and validated for the Portuguese Language: Epworth Sleepiness Scale and Pittsburgh Sleep Quality Index. The questionnaires were applied before and 6 months after surgical repair.

**Results:**

When analyzing the Epworth Sleepiness Scale, there was an improvement in sleep quality (p=0.0401). For the Pittsburgh Sleep Quality Index, only for sleep disorder there was improvement in quality of sleep after surgery (p=0.0127).

**Conclusion:**

Women with urinary incontinence, submitted to surgery with sling, showed improvement in both quality of sleep and sleep disorder.

## RESUMO

**Objetivo:**

Avaliar a qualidade do sono em mulheres com incontinência urinária antes e após correção cirúrgica do tipo *sling*.

**Métodos:**

Estudo prospectivo do tipo série de casos de mulheres com diagnóstico urodinâmico de incontinência urinária de esforço. Para avaliar a qualidade subjetiva do sono, utilizaram-se dois questionários específicos e validados para a língua portuguesa: Escala de Sonolência de Epworth e Índice de Qualidade do Sono de Pittsburgh. Os questionários foram aplicados antes e 6 meses após a correção cirúrgica.

**Resultados:**

Ao analisar a Escala de Sonolência de Epworth, houve melhora da qualidade do sono (p=0,0401). Para o Índice de Qualidade do Sono de Pittsburgh, apenas para o transtorno do sono, houve melhora da qualidade do sono após a cirurgia (p=0,0127).

**Conclusão:**

Mulheres com incontinência urinária, submetidas à correção cirúrgica por meio de *sling*, apresentaram melhora tanto na qualidade do sono como no transtorno do sono.

## INTRODUCTION

Sleep is a reversible behavior state of perception inhibition and relative unresponsiveness to the environment.^(^ ^1^ ^)^ The neurobiological processes that occur during sleep are necessary in virtually every species to maintain physical and cognitive health. Sleep disorders may hinder one's performance while studying, working and impact family and social relations, in addition to being associated to a higher risk of accidents, at work and behind the wheel.^(^ ^2^ ^)^

To adequately investigate sleep disorders, we can use clinical evaluations and objective and subjective measurements. Among the objective measures, polysomnography is a very important tool, for it allows the evaluation of both normal and altered sleep.^(^ ^3^ ^)^ Polysomnography is conducted during a night's sleep, with continuous monitoring of electrophysiological variables, such as electroencephalogram, ocular movements, thoracoabdominal movements, air flow, and submental muscle tone, to characterize sleep amount and quality. It also includes an electrocardiogram to record heart rate and rhythm, and measurement of arterial oxygen saturation.^(^ ^4^ ^)^ To conduct this exam, we need a facility with an adequate physical structure and professionals with specific training, which often restricts its use in the day to day life.^(^ ^5^ ^)^

The instruments for subjective measurements may be used in the clinical routine and also in research protocols.^(^ ^5^ ^)^ Some of them evaluate sleep in its general aspects, while others are directed more at certain alterations, such as those used to evaluate excessive daytime sleepiness (EDS). The Pittsburgh Sleep Quality Index (PSQI)^(^ ^6^ ^)^ provides an index of the nature and severity of sleep disorders; that is, a combination of quantitative and qualitative information about sleep. In turn, the Epworth Sleepiness Scale (ESS)^(^ ^7^ ^)^ was developed to evaluate the occurrence of EDS, taking into account the possibility of the individuals dozing off during daily life situations. This scale is widely used because it is considered simple, easy to understand, and quick to fill out.^(^ ^5^ ^)^

In its last publication, the International Continence Society defines urinary incontinence as all involuntary loss of urine.^(^ ^8^ ^)^ It is relatively common, and its prevalence varies from 5% in young women to 50% in older women.^(^ ^9^ ^)^ Stress urinary incontinence (SUI) is the most common urinary complaint among women, followed by urge incontinence, especially during menopausal transition.^(^ ^10^ ^)^

Common risk factors for SUI include advanced age; Caucasian race; obesity; vaginal births, when the passage of the fetus may damage local muscles and nerves; traumatic births that require a forceps or episiotomies; multiparity; pregnancy at an advanced age; estrogen deficiency; conditions associated to intra-abdominal pressure increase; smoking; diabetes; collagen diseases; neurological diseases; and previous hysterectomy.^(^ ^11^ ^)^

For women, in addition to hygienic discomfort, SUI causes social, sexual, psychic, and economic problems.^(^ ^12^ ^)^

Some symptoms associated to urinary incontinence affect sleep quality in women, causing, for example, nocturia (getting up from bed more than once per night to urinate) and nocturnal enuresis (involuntary urine loss while sleeping).^(^ ^13^ ^)^

Poor sleep quality in elderly individuals may be associated to nocturia, which is the most frequent cause of sleep disorders among institutionalized elderly individuals and affects 70% of this population.^(^ ^14^ ^)^

Among the treatment options available for this condition, the surgical approach offers the best results. Perineal exercises to strengthen pelvic floor muscles, perineal electrostimulation, and drugs, have all been suggested in the literature as treatment options, but their results are not very promising.^(^ ^15^ ^)^

The objective of SUI-correcting surgeries is to provide adequate suburethral support, reconstituting the function of muscular-fascial elements responsible for stabilizing the urethra during abdominal stress maneuvers.^(^ ^16^ ^)^

Slings have been used for almost 100 years to treat urinary incontinence in women. It is considered that, in addition to the support offered by the material placed in the suburethral region, the sling provides a structure over which a fibrosis process will develop. This structure will be the main long-term support element for the urethra, thus functionally substituting pubourethral and urethropelvic ligaments, whose functions are compromised in urethral sphincteric insufficiency.^(^ ^17^ ^)^

Studies investigating the possible implications of urinary incontinence in the quality of sleep of women with pelvic floor dysfunctions are still scarce.

## OBJECTIVE

To evaluate sleep quality in women with urinary incontinence before and after surgical correction with sling.

## METHODS

This study was conducted in accordance with the guidelines of Resolution 196/96 of the National Health Council (CNS - *Conselho Nacional de Saúde*) to ensure the rights and duties regarding the scientific community, the subjects of the study, and the State. This work was evaluated by the Research Ethics Committee of the *Faculdade de Medicina do ABC*, in Santo André, SP, Brazil, and approved under protocol 38.253, CAAE: 00800512.5.0000.0082. All patients received the proper information and signed an informed consent form.

This is a prospective case series study conducted with female patients with a urodynamic diagnosis of SUI. To evaluate the subjective amount of sleep, we used two specific questionnaires validated for the Portuguese language by Bertolazi:^(^ ^5^ ^)^ the ESS and the PSQI.

The PSQI^(^ ^18^ ^)^ evaluates the sleep quality of the previous month, and it is clinically useful in the evaluation of several sleep disorders that affect sleep quality.^(^ ^19^ ^)^ It includes 19 self-administered questions and 5 questions to be answered by bedroom companions. The latter are used for clinical information only. The 19 questions are grouped into 7 components, with weights distributed on a scale from 0 to 3: (1) subjective sleep quality; (2) sleep latency; (3) sleep duration; (4) habitual sleep efficiency; (5) sleep disorders; (6) use of sleeping medication; and (7) daytime dysfunction. Scores obtained from these components are added up to generate a global score between 0 and 21, in which the higher the score, the worst the sleep quality.

Epworth Sleepiness Scale^(^ ^20^ ^)^ was developed through the observation of the nature and occurrence of daytime sleepiness. It is a self-administered questionnaire about the likelihood of dozing off in daily situations. To graduate this likelihood of dozing off, patients use a scale from 0 to 3, in which 0 means ‘would never doze’ and 3 means ‘high chance of dozing’. Using a total score >10 as a cut-off point, it is possible to identify individuals with a high chance of having EDS.^(^ ^21^ ^)^ Total scores greater than 16 are indicative of severe somnolence, usually found in patients with moderate or severe obstructive sleep apnea syndrome, narcolepsy, or idiopathic hypersomnia.

The surgery used to treat SUI was the transobturator sling (Safyre T Plus^®^ Promedon^®^).

The questionnaires PSQI and ESS were applied by the principal investigator before and 6 months after surgical correction with sling. All participants included in the study were outpatients seen at the *Centro de Atenção à Saúde da Mulher* (CAISM) [Women's Health Center) in the city of São Bernardo do Campo (SP), of the *Faculdade de Medicina do ABC*, and were monitored between January and December of 2013.

After that, data were exported for statistical analysis using the software GraphPad Prism version 6.0. To compare the results of the pre- and post-surgery questionnaires we used the Mann-Whitney non-parametric test. The level of rejection of the null hypothesis was set at p=0.05 or 5%.

## RESULTS

The study included 36 female patients with a mean age of 48.2±6.8 years, and a urodynamic diagnosis of SUI. Patients who did not fill out the questionnaires in their entirety were excluded.

**Figure 1:**
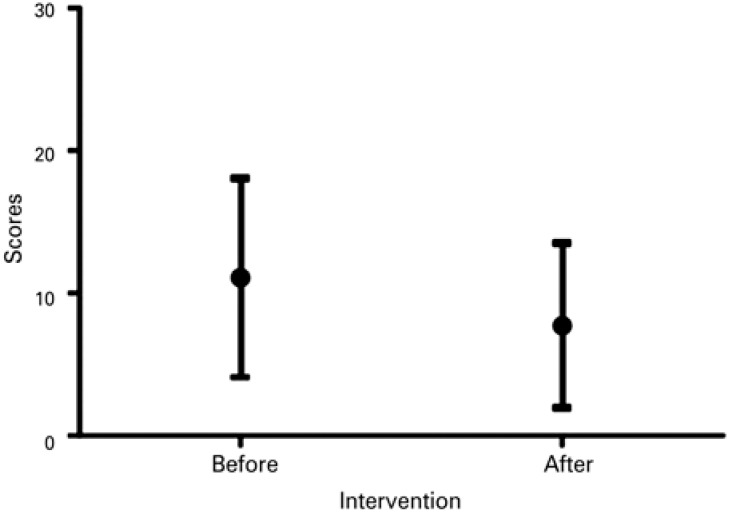
Median Epworth Sleepiness Scale score for sleep quality before and after surgery

When analyzing the ESS, we observed an improvement in sleep quality (p=0.0401) six months after the procedure to correct the SUI. The median scores before surgery and after surgery were 12 and 5.5, respectively, as shown in figure 1.

**Figure 2:**
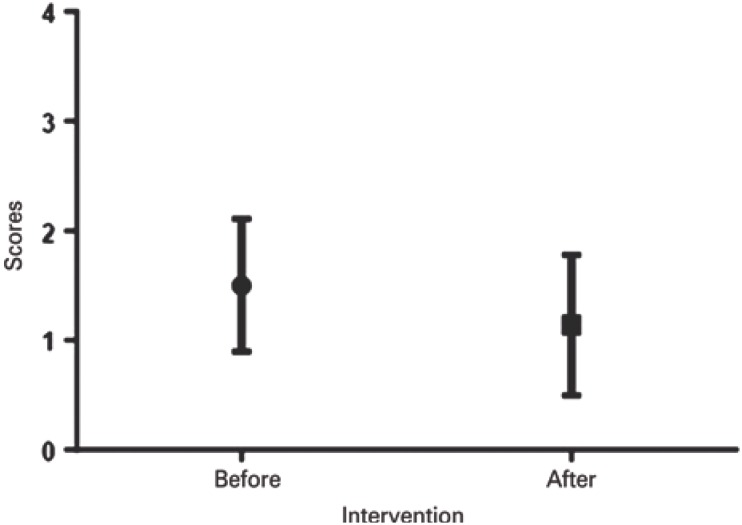
Median score before and after sling surgery, obtained through the Mann-Whitney non-parametric test, for the sleep disorder domain

We analyzed seven parameters (domains) of the PSQI. For the sleep disorder domain, we observed an improvement in sleep quality after surgery (p=0.0127). The median score before the sling surgery was 1.5, and after surgery, 1.0. Results are depicted in figure 2.

**Figure 3:**
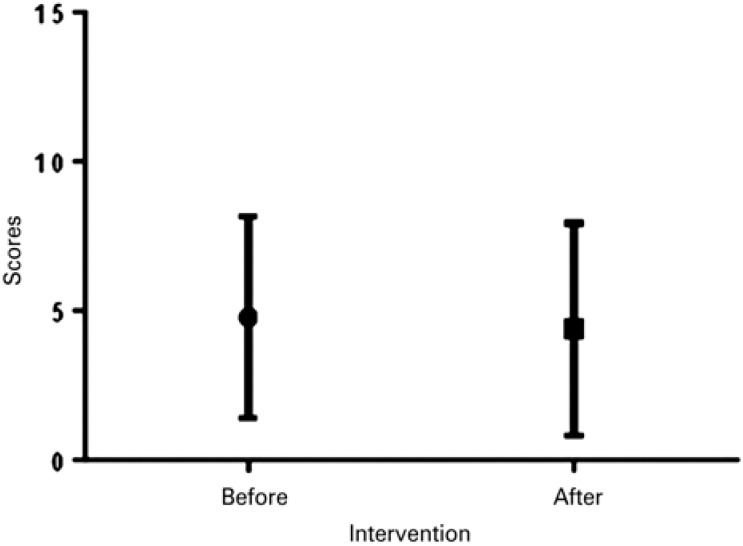
Total score of the Pittsburgh Sleep Quality Index obtained through the Mann-Whitney test

Regarding the evaluation of the other domains, there were no significant differences before and after surgery, and median score of 1.0 before and after the procedure: sleep duration (p=0.9740); sleep latency (p=0.9690); daytime dysfunction due to sleepiness (p=0.1739); sleep efficiency (p=0.8943); subjective sleep quality (p=0.4490); and use of sleeping medication (p>0.9999). Finally, the total score for the PSQI evaluation was p=0.3643. It was only regarding this last result that the median score before the sling surgery was 3.0 and after the surgery, 2.5. The behavior of this domain can be observed in figure 3.

## DISCUSSION

Sleep alterations may cause significant cognitive deficits, such as difficulty focusing and remaining attentive; memory loss; decreased strategic planning ability; motor function deficit; difficulty to control impulses; and diminished thought processing. These alterations, in addition to increasing the risk of accidents at work and behind the wheel, also hinder one's performance while studying and working and affect family and social relations.^(^ ^22^ ^)^

In a study conducted in the city of Lavras, State of Minas Gerais, Brazil, 73.3% of women presented poor sleep quality, and 20% already had sleep disorders. It is noteworthy that the population of the study comprised elderly women, and sleep is generally altered in this age group. Moreover, most of these individuals were on medications that could potentially affect sleep.^(^ ^14^ ^)^

A study that interviewed 1,424 elderly women found that 53% of them presented nocturia, 75% reported insomnia, and 71% reported a decrease in sleep quality. The authors also found a relation between nocturia and an increased frequency of daytime naps.^(^ ^23^ ^)^

All these alterations tend to cause early ageing and reduce life expectancy.^(^ ^23^ ^)^ The adoption of preventive measures by young adults is widely considered very important to prevent cardiovascular diseases, such as hypertension. Such measures include an improvement in life quality and sleep quality.^(^ ^24^ ^)^

Considering women with urinary dysfunction are susceptible to sleep disorders due to frequent visits to the bathroom to empty the bladder and/or change absorbent pads, it is very important to evaluate the sleep habits of these patients.

Understanding the sleep quality of women with SUI contributes to the development of educational activities and raises awareness about women's health. Many patients with SUI believe urinary loss is a normal part of ageing and that the options of treatment available do not improve the quality of life.

Data from this study clearly show that surgical correction can mitigate sleep dysfunctions in women with urinary incontinence. Surgery improves urinary loss and helps preventing diseases of high prevalence in women with sleep disorders, especially cardiovascular diseases.

## CONCLUSION

For women with urinary incontinence, the sling surgery led to better results regarding quality of sleep (assessed by Epworth Sleepiness Scale) and sleep disorders, such as insomnia, obstructive sleep apnea syndrome, and snoring (evaluated by the Pittsburgh questionnaire).
